# Oval Type of Human Mandibular Condyle in Panoramic Radiographs of a Tertiary Care Centre: A Descriptive Cross-sectional Study

**DOI:** 10.31729/jnma.7416

**Published:** 2022-06-30

**Authors:** Samarika Dahal, Alok Atreya, Sanjay Prasad Gupta, Srikant Natarajan

**Affiliations:** 1 Department of Oral Pathology and Forensic Dentistry, Maharajgunj Medical Campus, Institute of Medicine, Maharajgunj, Kathmandu, Nepal; 2 Department of Forensic Medicine, Lumbini Medical College, Tansen, Palpa, Nepal; 3 Department of Orthodontics and Dentofacial Orthopedics, Maharajgunj Medical Campus, Institute of Medicine, Maharajgunj, Kathmandu, Nepal; 4 Department of Oral Pathology and Microbiology, Manipal College of Dental Sciences, Mangalore, Karnataka, India

**Keywords:** *condyle*, *mandible*, *Nepal*, *X-ray*

## Abstract

**Introduction:**

Mandibular condyle is a prominent structure in the mandible, which forms the temporomandibular joint. An oval-shaped condyle is considered to be present with a normal temporomandibular joint and any morphological variation of the condyle is assumed to be pathologic in temporomandibular disorders. The aim of the study was to find out the prevalence of oval shaped mandibular condyle among orthopantomogram radiographs of patients visiting the tertiary care centre.

**Methods:**

A descriptive cross-sectional study was performed among 752 condyles visiting a tertiary care center from November 29, 2021 to April 1, 2022. The ethical approval was taken from the Institutional Review Committee (Reference number: 184 (6-11) 078/079) before conducting the study. Convenience sampling was done. The radiographs were first examined and the observed morphological type of mandibular condyle was noted. Data analysis was done using Statistical Package for the Social Sciences version 22.0. Point estimate at 95% Confidence Interval was calculated along with frequency and proportion for binary data.

**Results:**

The prevalence of oval shaped mandibular condyles out of 752 condyles was 416 (55.32%) (51.77-58.87 at 95% Confidence Interval). The oval-shaped condyle on the right side was 205 (54.52%) and on the left side was 211 (56.12%).

**Conclusions:**

The prevalence of oval shaped condyles among patients in this study was similar to the studies done in similar settings.

## INTRODUCTION

The mandible is a unique and dynamic bone that connects to the skull via the Temporomandibular Joint (TMJ).^1,2^ The mandibular condyle an integral component of TMJ extends superiorly toward the concave glenoid fossa of the temporal bone on each side, resulting in a bilateral craniomandibular articulation.^2-4^

Very few studies have investigated the shape of the mandibular condyle in the Nepali subset population. The oval-shaped condyle is considered to be present with a normal TMJ, and any morphological variation of the condyle is assumed to be pathologic. The findings of the study can provide a valuable insight to the clinicians to rule out the pathological alteration of the condyle from the anatomical variation.

The aim of the study was to find the prevalence of the oval type of mandibular condyle in patients visiting a tertiary care centre.

## METHODS

A descriptive cross-sectional study was conducted at the Department of Oral Pathology and Forensic Dentistry from November 29, 2021 to April 1, 2022. The ethical approval was taken from the Institutional Review Committee, Institute of Medicine, Maharajgunj, Kathmandu, Nepal (Reference number: 184 (6-11) 078/079). The Orthopantomogram (OPG) X-rays of patients visiting a tertiary care center aged 4 to 45 years free of any projection errors, showing a complete view of condyle on either side with optimum density and contrast were included in the study. All the radiographs with temporomandibular joint dysfunction, occlusal discrepancy, pathological and developmental abnormalities, history of trauma in the maxillofacial region, and edentulous patients were excluded from the study. Convenience sampling was done and the sample size was calculated according to the formula:


$$\begin{array}{ll} \text{n} & ={\text{Z}}^{2}\times \frac{\text{p}\times \text{q}}{{\text{e}}^{\text{2}}}\\ & =1.96^{2}\times \frac{0.68\times 0.32}{0.05^{2}}\\ & =335 \end{array}$$

Where,

n = minimum required sample size
Z = 1.96 at 95% Confidence Interval (CI)
p = prevalence of oval shaped mandibular condyle, 68%^5^
e = margin of error, 5%

Since convenience sampling was done, the sample size was doubled to 670. As one person has two mandibular condyles, we included data of 376 individuals comprising 752 mandibular condyles in the study.

The identification of the oval shaped mandibular condyle was based on the radiographic appearances by using panoramic radiographic technique.^6^ The radiographs were first examined with the built-in magnifying lens on the MacBook Pro. A screenshot of the X-rays were taken which were then saved as an image file with an identification code. These images were then imported as individual slides on Microsoft Powerpoint, the title of the slide being the identification code. These powerpoint slides were shared between the two authors for observing the morphological type. The radiographic images were then used to sketch the condylar contour along its radiolucent outline using curve lines and connectors, which could be accessed by clicking shapes on Microsoft powerpoint's home button. The observed morphological type of mandibular condyle was noted.

Data were entered in a Microsoft Excel 2016 and analyzed using Statistical Package of for the Social Sciences version 21.0. Point estimate at 95% CI was calculated along with frequency and proportion for binary data and mean and standard deviation for continuous data.

## RESULTS

The prevalence of oval shaped mandibular condyles out of 752 condyles was 416 (55.32%) (51.77-58.87 at 95% Confidence Interval). The frequency of ovalshaped condyle on the right side was 205 (54.52%) and on the left side was 211 (56.12%) (Table 1).

**Table 1:** Site of involvement of xanthelasma palpebrarum (n = 64).

Side of condyles	Oval condyles n (%)
Right condyle	205 (54.52)
Left condyle	211 (56.12)

The study included panoramic radiographs of 376 individuals, resulting in a total of 752 mandibular condyles. Out of the 376 individuals, 208 (55.32%) were females and 168 (44.68%) were males with a female-to-male ratio of 1.23:1. The patients were aged between 4 to 45 years. The mean age of the patients was 17.56±6.22 years.

The oval shaped condyle was seen in 168 (44.68%) males and 208 (55.32%) females respectively. In males, the frequency of oval right mandibular condyle was 96 (57.14%) and oval left mandibular condyle was 98 (58.33%). Similarly, in females, its frequency was 109 (52.40%) and oval left mandibular condyle was 113 (54.33%) (Table 2).

**Table 2:** Sex-wise distribution of oval shaped condyle (n = 416)

Sex	Left condyle n (%)	Right condyle n (%)
Male	98 (58.33)	96 (57.14)
Female	113 (54.33)	109 (52.40)

## DISCUSSION

In our study, the prevalence of oval shaped mandibular condyles was 55.32% where 54.52% of them was found on the right side and 56.12% was on the left side. The mandibular condyle morphology was first studied in 1966.^7^ Many studies then followed through since they used diverse nomenclature, however, the morphology defined and classified corresponded to each other.

The various qualitative and quantitative analyses of mandible based on gonial angle, condylar and coronoid heights, bigonial width, coronion-gonion distance, condylar volume, and the condylar surface have been utilized for estimating age, sex, and ethnicity in diverse groups.^3,5,8-12^ However, very few studies have inspected the shape of the mandibular condyle with potential implications in clinical diagnosis and therapeutic planning. Although Computed Tomography (CT) scan and ultrasonography techniques are utilized for studying mandibular condyle morphology,^11,13,14^ panoramic radiography is one of the most often utilized low-cost methods.^4,6,15^

The oval shape defined in this present article has a round or oval superior surface. It is reported to be the most prevalent with similar distribution on right and left sides similar to our study.^3^ Round or oval type was the most frequent in both sexes similar to other studies conducted in populations of Brazil, Iraq, India, and Pakistan.^5,6,15-17^ However, lower than the Bangladeshi population. A study based on the Pakistani population reported oval condylar morphology to be prevalent more among males.^17^ While Bangladeshi population-based study had higher frequency.^5^

The gender, age, facial type, functional load, occlusal force, and malocclusion, all influence condylar morphology.^18-20^ The reason for the condyle to have an oval shape as the predominant type in our study could be attributed to the younger age of the sample taken for the study. The wear of the condylar head, which leads to osteophyte growth, can cause the shape of the condyle to change in older age groups due to the continual functional loading to which the condyle is subjected.^21,22^ Many studies have reported the shift in shape from oval to different shapes as people become older.^21,23,24^ It can be a sign that degenerative joint disease is more common among the elderly.

The cortical bone in condyles of individuals less than 40 years of age has a smooth, regular, convex shape, but deterioration and a notched irregularity of the cortical bone plate become prevalent with the increasing age.^23^ After the seventh decade, the morphological alteration of condyles may occur to a polygonal or flattened shape. As the radiographs included in our study were mostly of sub adults less than 40 with all the teeth present in the OPGs explaining the reasons for the prevalent oval type of the condyle.^20^

The single-centre nature of the present study could not be generalized to the whole Nepalese population. Further studies comparing the OPGs obtained at a young age with that of the old age of the same individual may help to study the change in such morphological patterns.

## CONCLUSIONS

The prevalence of the oval type of the mandibular condyle was similar to other studies done in similar settings. These findings might be helpful for clinical considerations.
